# RETRACTION: Injectable Genetic Engineering Hydrogel for Promoting Spatial Tolerance of Transplanted Kidney in Situ

**DOI:** 10.1002/advs.76182

**Published:** 2026-06-15

J. Lin, S. Liu, X. Xue, J. Lv, L. Zhao, L. Yu, H. Wang, and J. Chen, “Injectable Genetic Engineering Hydrogel for Promoting Spatial Tolerance of Transplanted Kidney in Situ,” *Advanced Science* 11, no. 48 (2024): 2408631, https://doi.org/10.1002/advs.202408631.

The above article, published online on 5 November 2024 in Wiley Online Library (http://onlinelibrary.wiley.com/), and its correction (https://doi.org/10.1002/advs.74915), have been retracted by agreement between the journal Editor‐in‐Chief, Kirsten Severing; the deputy editor, Yuming Hu; and Wiley‐VCH GmbH.

A third party notified the journal that rheological data in Figure 1 and in Supplementary Figures 4, 5A, and 5C of this article are nearly identical to rheological data published in an earlier article by different authors [Figures 1, 2, and supplemental figures in Correa et al. 2022 (https://doi.org/10.1016/j.matt.2022.03.001)]. Additionally, the approach and methods used in the above article are extremely similar to those described in Correa et al., which is not cited as a foundational work in the above article. The editors and publisher recognized this overlap in content and methodology as problematic and were prepared to retract the article, in keeping with publisher and COPE guidelines.

During the process of retraction, a correction submitted by the authors was published in error. The correction does not change the fundamental concerns that the editors and publisher have with the original publication. Because of the concerns about overlapping data and methodology in the version of record for this article, the editors have lost confidence in the originality of the results reported. Therefore, it must be retracted.

